# Influence of cell bioenergetics on host-pathogen interaction in the lung

**DOI:** 10.3389/fimmu.2025.1549293

**Published:** 2025-04-03

**Authors:** Gaurav Kumar Lohia, Sebastián A. Riquelme

**Affiliations:** Department of Pediatrics, Columbia University, New York, NY, United States

**Keywords:** immunometabolism, MACROPHAGE METABOLISM, itaconate, ESKAPE bacteria, fungal infection, host-pathogen interaction, bioenergetics, pneumonia (infectious disease)

## Abstract

Pulmonary diseases, arising from infections caused by bacteria, fungi, and viruses, or stemming from underlying genetic factors are one of the leading causes of mortality in humans, accounting for millions of deaths every year. At the onset of pulmonary diseases, crucial roles are played by phagocytic immune cells, particularly tissue-resident macrophages, in regulating the immune response at the mucosal barrier. Recent strides have illuminated the pivotal role of host bioenergetics modulated by metabolites derived from both pathogens and hosts in influencing the pathophysiology of major organs. Their influence extends to processes such as the infiltration of immune cells, activation of macrophages, and the polarization phenomenon. Furthermore, host-derived metabolites, such as itaconate, contribute to the promotion of anti-inflammatory responses, thereby preventing immunopathology and facilitating the preservation of mucosal niches to thrive for the long-term. This review explores recent advancements in the field of immunometabolism, with a particular emphasis on the intricacies of disease progression in pulmonary infections caused by bacteria such as *P. aeruginosa, M. tuberculosis* and *S. aureus* and fungi like *C. albicans.*

## Introduction

According to recent epidemiology studies, pulmonary infections caused by pathogens such as *Pseudomonas aeruginosa*, *Mycobacterium tuberculosis*, *Staphylococcus aureus* and *Candida albicans* represent significant contributors to global mortality (1, 2). These infections are frequently observed in individuals with co-morbidities, such as cystic fibrosis (CF), chronic obstructive pulmonary disease (COPD), and primary ciliary dyskinesia (PCD), as well as during ventilator-associated pneumonia (VAP), which complicate prognosis and disease management (3–9). In the lung, these pathogens trigger a brisk inflammatory response that often aids in infection clearance. However, in specific contexts, this immune response is either insufficient or aberrant, leading to tissue damage, pathogen persistence, and chronic colonization. Multiple immunomodulatory platforms, including metabolites, cytokines, pathogen-associated molecular patterns (PAMPs) and damage-associated molecular patterns (DAMPs) influence the outcome of the immune response (10). Despite advances in understanding these mediators, their role in modulating mucosal integrity through host bioenergetics remains an emerging area of study. This review examines recent progress in immunometabolism research, with particular emphasis on host bioenergetics during pulmonary infection by these pathogens.

### Host bioenergetics and organ homeostasis

Host cell primarily rely on two pathways for producing energy; namely, glycolysis and oxidative phosphorylation (OXPHOS). These routes provide cells with ATP and substrates for various cellular processes. However, different cell types specialize in specific pathways to maintain their bioenergetic integrity. For example, neurons rely heavily on OXPHOS as they lack key components of the glycolytic pathway (11). Similarly, heart generates ~90% of its energy from this same mitochondrial platform (12). Red blood cells, on other hand, use glycolysis to synthesize ATP, as they lack mitochondria (13). Brown adipose tissue (BAT), which is involved in thermogenesis, uses the energy generated from fatty acid oxidation (FAO) in mitochondria to produce heat instead of ATP (14, 15). In contrast, immune cells, such as macrophages exhibit remarkable plasticity in their bioenergetic pathways. This bioenergetic dynamics plays a crucial role in regulating their immunological properties. Pro-inflammatory responses in macrophages are predominantly associated with enhanced glycolysis and impaired OXPHOS (16). Consistently, anti-inflammatory responses are marked by functional OXPHOS and many networks that fuel this platform, such as FAO and the tricarboxylic acid (TCA) cycle (17). During infection, balanced pro- and anti-inflammatory responses are critical in macrophage effector activity, as it helps in appropriate disease resolution, thereby maintaining tissue integrity (18, 19). Thus, glycolysis and OXPHOS are major coordinators of host cell bioenergetics, and their modulation during infection might define the outcome of diseases.

## Host bioenergetic reprogramming during infection

The lung serves as the primary site of infections caused by *P. aeruginosa*, *M. tuberculosis*, and *S. aureus*. This mucosal environment is guarded by immune cells, including resident subsets like alveolar macrophages and other infiltrating phagocytes, such as neutrophils and monocytes. During infection, these myeloid cells undergo metabolic reprogramming to bolster their inflammatory responses. This process is coordinated by many PAMPs, such as lipopolysaccharide (LPS), flagella, and peptidoglycan, which, respectively, interact with Pattern Recognition Receptors (PRRs), like Toll-like receptor 4 (TLR4), TLR5, and hexokinase (HK) (10, 20). These PAMP-PRR interactions alter host bioenergetics, particularly by disrupting OXPHOS. OXPHOS dysfunction is further exacerbated by the downregulation of key TCA cycle enzymes, like isocitrate dehydrogenase (IDH), which is essential for the generation of metabolites that sustain OXPHOS activity, such as αketoglutarate (21). To overcome this bioenergetic deficit, infected cells switch to glycolysis (22, 23). This shift not only provides ATP to maintain cell viability but also stabilizes hypoxia-inducible factor 1-alpha (HIF1α). Once stabilized, HIF1α translocates to the nucleus and orchestrate the expression of a range of pro-inflammatory cytokines, which are critical for confronting the infection (24). However, if not adequately controlled, excessive inflammation can result in tissue damage, a condition often observed in inflammatory pathologies like sepsis, COVID-19, influenza, lupus, and arthritis (3, 25, 26). In such situations, tight regulation of bioenergetic pathways becomes crucial to balance inflammation and prevent organ oxidation. Thus, during infection, appropriate bioenergetic reprogramming of myeloid cells ensures effective immunity compatible with host health.

In this review, we will examine evidence on the impact of bioenergetics on immune regulation and bacterial persistence during infection caused by (**A**) intracellular pathogen such as *M. tuberculosis* and (**B**) extracellular pathogen such as *P. aeruginosa* and *S. aureus*. Furthermore, (**C**) we will cover emerging information pertinent to fungal pathologies, and how specific the survival of these organisms is modulated by metabolic cues.

### A. Intracellular pathogens

#### Macrophage metabolism shapes *M. tuberculosis* infection

*M. tuberculosis* (herein Mtb), the causative agent of tuberculosis (TB), remains a major global health challenge, with nearly a quarter of the world’s population harboring latent infections (Global tuberculosis report 2024) (27). Mtb transmission primarily occurs through the inhalation of aerosolized droplets containing the pathogen, which subsequently deposit in the pulmonary alveoli (28). Mtb primarily infects alveolar macrophages, which are expected to be the primary cellular barrier against the infection (28, 29). Mtb can also subsist within a range of other different phagocytes, including neutrophils and dendritic cells, as well as non-immune cells such as fibroblasts, endothelial cells, and hematopoietic stem cells (30, 31). The infection triggers immune cell recruitment, leading to the formation of granulomas (32), where Mtb evades clearance by suppressing antigen presentation and autophagy (33, 34). The granuloma environment determines whether the infection remains contained or progresses to systemic disease.

**Figure 1:**
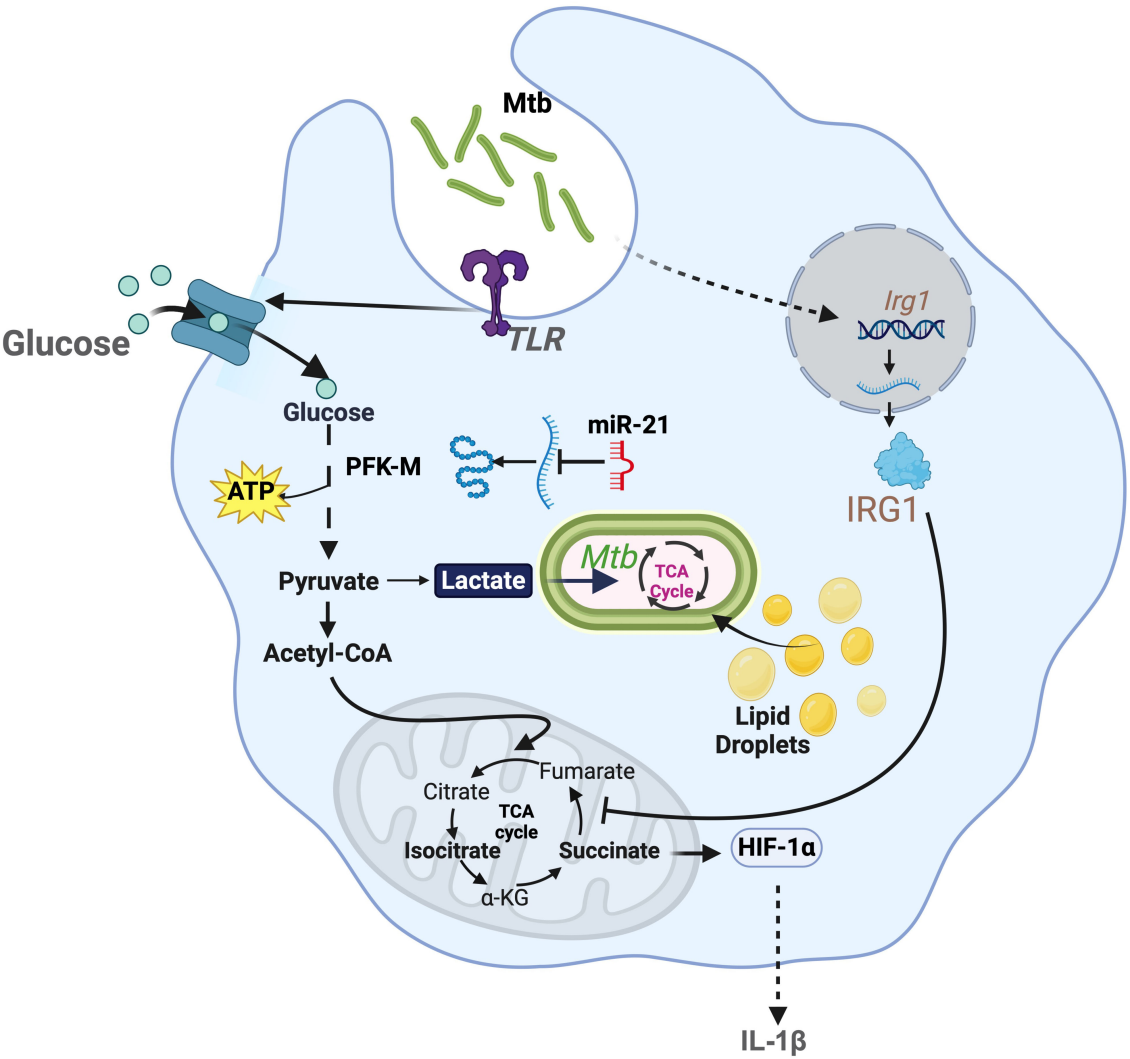
Modulation of bioenergetic pathways by *M. tuberculosis*: *M. tuberculosis* induces robust glycolysis in myeloid cells by increasing the uptake of glucose via TLR signaling. This also promotes accumulation of lactate, which is used by the pathogen to generate energy. Lactate also hinders *M. tuberculosi*s growth. In response to this, *M. tuberculosis* induces expression of miR-21, that regulates the expression of phosphofructokinase-M (PFK-M). *M. tuberculosis* regulates host TCA cycle by promoting the expression of *Irg1*, which inhibits SDH. This metabolic control attenuates the expression of pro-inflammatory cytokine, such as IL-1β.

Mtb survival relies on host macrophage glycolysis. Emerging evidence indicates that different Mtb strains influence macrophage glycolysis to persist. For example, many strains like H37Ra (avirulent strain) and H37Rv (virulent strain) trigger glycolysis, which seems to be linked to TLR2 stimulation (35). This TLR2 activation drives the expression of the glucose transporters GLUT6 and GLUT1/3 (36) (**Figure 1**). ESAT-6, a 6kDa early secretory antigenic target protein, enhances macrophage glucose uptake by facilitating GLUT3-mediated transport (36, 37). Multidrug-resistant (MDR) strains exhibit elevated ESAT-6 expression, further augmenting host cell glucose uptake (38). Mtb uses the glycolytic byproduct lactate as a major carbon source to fuels its TCA and gluconeogenesis. This is essential for the bacterium to proliferate, as Δ*lldD2* strains, which cannot oxidize lactate, fail to grow intracellularly in human macrophages (39). However, other groups have reported opposite results, where lactate enhances Mtb clearance (40). To avoid their eradication, these Mtb strains inhibit host glycolysis through microRNA-21 (miR-21), which targets phosphofructokinase-M (PFK-M) (41) (**Figure 1**). Furthermore, other Mtb strains differentially regulate downstream glycolytic genes, such as 6-phosphofructo-2-kinase/fructose-2,6-biphosphatase 3 (*Pfkfb3*) (42). Differential regulation of glycolysis by Mtb is mediated by strain-specific virulence factors. For instance, less virulent strains, such as Mtb CDC1551, induce significantly higher levels of *Pfkfb3* compared to the hypervirulent Mtb HN878 (42, 43). Consistently, drug-resistant strains like W_7642 exhibit even lower *Pfkfb3* expression than HN878. This regulation is linked to variations in cell wall lipid composition, particularly PDIMs (phthiocerol dimycocerosates) and other lipids that are highly expressed in MDR strains (42, 44). HIF-1α, which regulates the transcription of glycolytic genes, requires nitric oxide (NO) for its activity (45). However, MDR and extensively drug-resistant strains of Mtb induce an augmented IL-10 response, which inhibits NO production. This disruption impairs glycolysis induction (46, 47). These findings highlight the adaptability of Mtb strains in calibrating host glycolysis to sustain survival.

Mtb also disrupts the macrophage TCA cycle to persist (43). Mtb infection downregulates the enzymes IDH2 and succinate dehydrogenase (SDH). While it still remains unclear how Mtb suppresses SDH function, this pathogen limits IDH2 activity by stimulating TLR2 signaling and downregulating the deacetylase SIRT3 (48). Collectively, these interferences break the TCA cycle, resulting in the accumulation of isocitrate and succinate, respectively (21, 49) (**Figure 1**). This process repurposes mitochondria from producing energy to manufacture ROS (3). During early phase of infection, Mtb exploits ROS to enhance its replication and induce macrophage necrosis (50). This is facilitated by the generation of reactive nitrogen species (RNS), which act as potent inhibitors of the electron transport chain (ETC), further aggravating ROS signaling (51–53).

Mtb infection drives itaconate synthesis, which also contributes to pathogenesis (**Figure 1**). By stimulating the TLR2-STING axis, Mtb facilitates host upregulation of the enzyme *Irg1* (Immunoresponsive Gene 1, also known as Aconitate decarboxylase 1; ACOD1) (54). This enzyme diverts TCA cycle aconitate to generate itaconate (17). Itaconate drives immunosuppression, maintaining host survival and thus providing Mtb with a functional niche to thrive for the long-term. Indeed, mice lacking *Irg1* rapidly succumb to the infection, process mediated by neutrophil-driven inflammation (55). Furthermore, Mtb prospers by exploiting lipids and fatty acids as key nutritional sources. Mtb induces abnormal lipid accumulation in macrophages, leading to the formation of foamy cells (56). These metabolically altered cells exhibit elevated expression of genes involved in lipid synthesis and uptake, while showing reduced expression of genes responsible for lipid efflux (56). Mtb worsens this setting by inhibiting lipid droplet degradation, particularly by suppressing lipophagy, thereby creating a favorable environment for bacterial growth (56–58).

Collectively, these findings suggests that Mtb survival hinges on its ability to manipulate host macrophage metabolism. By reprogramming glycolysis, disrupting the TCA cycle, and exploiting lipid metabolism, Mtb outcompetes immune responses, sustains its growth, and establishes a persistent niche within the host.

### B. Extracellular pathogens

#### *P. aeruginosa* exploits pro-OXPHOS metabolites to thrive in the host

*P. aeruginosa*, a Gram-negative ESKAPE pathogen, induces a brisk inflammatory response in the respiratory tract. Instead of clearing the infection, this inflamed milieu aggravates tissue destruction, providing *P. aeruginosa* with cell debris and metabolites to flourish. This environment supports bacterial growth, with the pathogen existing as planktonic cells or biofilms (59). Macrophage metabolic reprogramming plays a central role in this process, as the succinate released from this cell, a pro-OXPHOS nutrient, fuels *P. aeruginosa’s* TCA cycle and bioenergetics (59). As directed by the catabolite repressor locus (*Crc*), succinate is the preferred carbon source for *P. aeruginosa*, which is consumed before any other nutrient available (59–61). Thus, the survival of *P. aeruginosa* in the alveolar space is tightly linked to host OXPHOS, primarily to succinate metabolite activities.

*P. aeruginosa* is a prominent pathogen in people with CF (pwCF) (19). CF is caused by mutations in the CF transmembrane conductance regulator (CFTR) (62). In pwCF, lack of CFTR compromises the function of PTEN, which is an essential metabolic checkpoint that directs mitochondrial bioenergetics. At baseline, CFTR forms a complex with PTEN at the cell membrane, which directs mitochondrial OXPHOS (62). In pwCF, lack of the CFTR-PTEN complex drives mitochondrial OXPHOS disruption, leading to succinate accumulation and release (62). During infection, this succinate supports *P. aeruginosa* bioenergetics, promoting bacterial proliferation, stimulation of inflammation, and massive infiltration of myeloid cells (59, 62). This succinate-rich milieu worsens IL-1β-driven inflammation, facilitating alveolar oxidation and progressive pulmonary decline (62).

**Figure 2:**
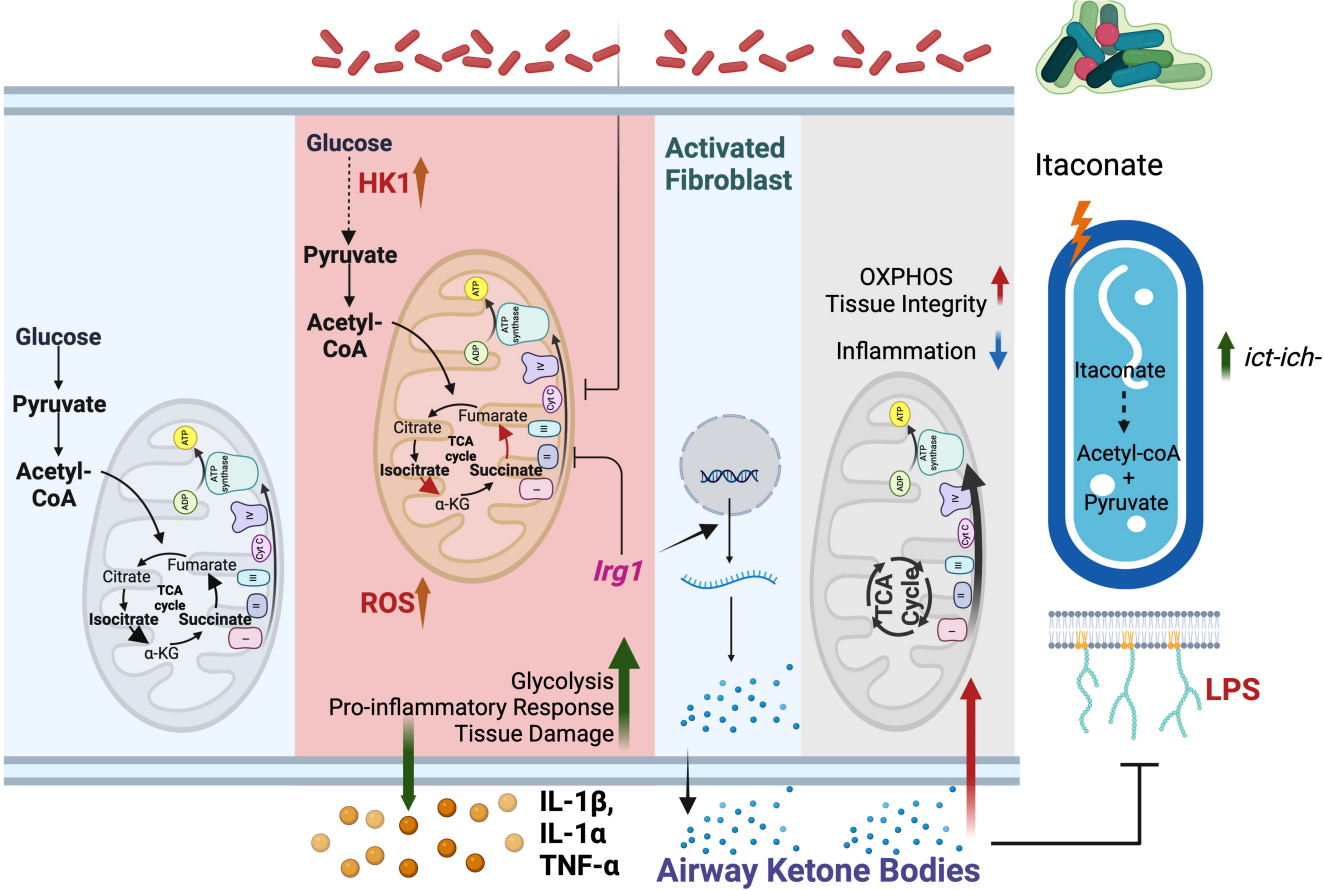
Mitochondrial metabolites drive *P. aeruginosa* infection. Left panel: At baseline, macrophage bioenergetics is primarily fueled by OXPHOS. Glycolysis contribution to ATP synthesis is limited. Middle panel: Upon *P. aeruginosa* infection, OXPHOS is impaired, and glycolysis is increased. Glycolysis drives release of tissue-damaging cytokines. To mitigate immunopathology, mitochondria express *Irg1* and produces itaconate. Right panel: *P. aeruginosa* adapts to itaconate by increasing the *ict-ich-ccl* locus, selecting for strains that exploit the immunometabolite to generate energy. Furthermore, itaconate-adapted *P. aeruginosa* also favor local enrichment of ketone bodies, particularly from fibroblasts. These ketone bodies maintain tissue integrity by suppressing *P. aeruginosa* expression of surface-exposed LPS and by fueling host OXPHOS. This milieu favors the progressive establishment of infection tolerance.

*P. aeruginosa* exacerbates host bioenergetic reprogramming and succinate release by exposing LPS on its surface through the transporter *lptD* (63). By stimulating TLR4, membrane-attached LPS disrupts lung mitochondrial OXPHOS, impairing host bioenergetics (10, 64) (**Figure 2**). Mechanistically, LPS depletes pulmonary ATP synthase, compromising OXPHOS integrity. In response, respiratory cells upregulate glycolysis, fueling the release of pro-inflammatory cytokines that accelerate tissue damage, such as IL-1β and elevated bacterial burden (3, 19). Furthermore, this inflamed environment permeabilizes the alveolar space, enabling the infiltration of different nutrients from circulation that the pathogen exploits to reduce LPS exposure and thus adapt to the host to be better tolerated, like ketone bodies (19) (**Figure 2**).

Macrophage succinate metabolism also drives ROS production through SDH and IDH (65). However, by sensing this ROS, *P. aeruginosa* activates the bacterial antioxidant response, limiting planktonic growth and fostering the formation of biofilms (62, 66). This lifestyle shift permits the bacterial community to persist in harsh environments, such as the imparted by antibiotics (67–69). This setting has been observed in CF, COPD, PCD and VAP, validating the clinical significance of succinate and ROS metabolism during *P. aeruginosa* pulmonary pathology.

#### *P. aeruginosa* adapts to itaconate to capitalize persistent infection

To balance the harmful effects of succinate, LPS, and ROS, macrophages produce itaconate as a protective immunometabolite (70). However, *P. aeruginosa* turns this host defense into an advantage. In contrast with most opportunist, *P. aeruginosa*’s genome harbors the *ict-ich-ccl* locus (71). This platform helps the pathogen to breakdown itaconate into less toxic metabolites, like acetyl-CoA and pyruvate, which enter the bacterial central metabolism to drive bioenergetics (59, 71). Furthermore, *P. aeruginosa* adapts to the outer membrane stress caused by itaconate by forming biofilms, gaining protection against diverse threats, such as antibiotics, ROS, antibodies, and phagocytosis (59) (**Figure 2**). As reported in pwCF, throughout the course of the infection, *P. aeruginosa* adapts to this itaconate-rich environment, selecting for communities that co-evolve with the immunometabolite (59). These strains further exploit itaconate to enrich pulmonary ketone bodies, which promote bacterial populations that are better tolerated by the host immune system (19) (**Figure 2**). By inducing infection tolerance, these ketone-adapted *P. aeruginosa* communities attenuate immunopathology, preserving both host survival and the lung as a functional niche to thrive for the long-term (19).

The extraordinary ability of *P. aeruginosa* to survive in mucosal tissues synchronized with the bioenergetic changes experience by infected macrophages is strong evidence of co-evolution with host defenses. This principle is supported by the development of infection tolerance, where *P. aeruginosa* readily adapts to the airway environment integrating into the local microbiome as discrete communities to thrive. These findings highlight the clinical significance of host bioenergetics directing chronic pathogen activities in mucosal tissues.

#### *S. aureus* adapts to host bioenergetic reprograming to subsist

**Figure 3:**
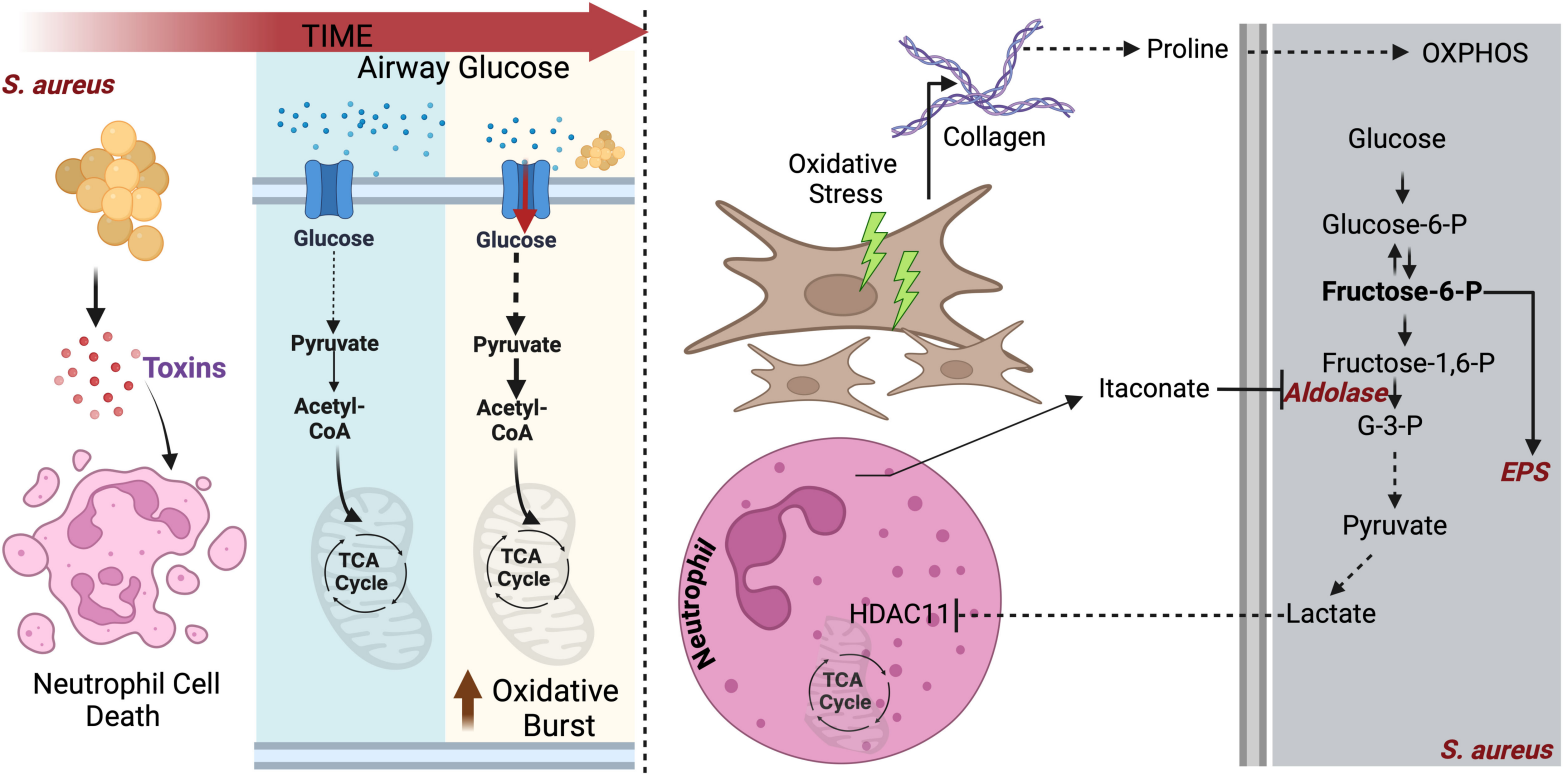
*S. aureus* adapts to lung bioenergetic changes: Left panel: During initial infection, *S. aureus* causes pulmonary pathology by secreting various toxins that kills neutrophils. To maintain survival, neutrophils deplete glucose to fuel their glycolysis, which is required to eliminate the pathogen via oxidative burst. Right panel: In addition to consume glucose, infected neutrophils block *S. aureus* glycolysis by inhibiting aldolase. Glycolysis interference by neutrophils promotes *S. aureus* adaptation to proline, released from activated fibroblast. Proline fuels *S. aureus* OXPHOS.

*S. aureus* is a Gram-positive ESKAPE pathogen that normally resides in the human microbiome (72). Under still unclear conditions, it can invade mucosal tissues, causing severe and life-threatening infections such as endocarditis, bacteremia, and pneumonia (72). The risk escalates when medical devices become contaminated, providing a gateway for infection (72, 73). In infected subjects, *S. aureus* drives both acute and chronic infections, characterized by intense neutrophil-mediated inflammation (74, 75). To enhance tissue damage and neutrophil infiltration, *S. aureus* produces toxins like leukocidins and hemolysins, which induce host cell death and create an inflammatory, oxidative environment (76, 77) (**Figure 3**). Instead of being cleared, *S. aureus* thrives in this detrimental milieu, feeding off nutrients derived from damaged tissue.

The preferred *S. aureus* carbon source is glucose, which is used to drive glycolysis (78, 79). However, in the host lung, glucose is limited, as myeloid cells consume it through their own glycolysis in response to *S. aureus* sensing (78–80). This glucose limitation forces *S. aureus* to adapt to alternative carbon sources to persist. During pneumonia, tissue repair mechanisms activate fibroblasts to release proline, a key component of collagen (81, 82). *S. aureus* adapts to utilize proline, particularly in diseases like CF, where chronic infection and progressive tissue scarring result in extensive collagen deposition (82). By capitalizing on proline availability during tissue remodeling, *S. aureus* generates energy and biofilms to sustain infection, promoting inflammation and persistent infection (82) (**Figure 3**). The high metabolic plasticity harbored by *S. aureus* provides the pathogen with essentials advantages to coexist with metabolically reprogrammed phagocytes.

Host cells respond to *S. aureus* infection by releasing itaconate (83). However, unlike *P. aeruginosa*, *S. aureus* lacks the *ict-ich-ccl* locus and hence cannot degrade itaconate (83). To persist in the itaconate-rich airway, *S. aureus* forms biofilms. To generate these communities, *S. aureus* specializes in the manufacture of extracellular polysaccharides (84, 85) (**Figure 3**). Furthermore, this pathogen exploits this same itaconate response to abrogate neutrophil function, particularly by blocking the oxidative burst required to kill the bacteria. In tissues different than the lung, *S. aureus* releases lactate, which contributes to immune evasion by altering the activity of Histone deacetylase 11 (HDAC11), redirecting host cells to produce IL-10 and thus skew immune responses towards an immunosuppressed phenotype (86) (**Figure 3**).

By adapting to glucose scarcity and other pulmonary amino acids involved in repair, like proline, *S. aureus* generates energy to survive. Furthermore, by exploiting the own host metabolic response to preserve tissue integrity, itaconate, *S. aureus* disrupt neutrophil function, skewing immune responses towards a state of infection tolerance. These findings, phenocopying the behavior of other pathogens like Mtb and *P. aeruginosa*, showcase the ability of *S. aureus* to thrive in bioenergetically altered environments, like the inflamed lung.

### C. Fungal infections

#### Metabolic pathways driving fungal infection dynamics

Fungal infections, primarily caused by *Candida albicans, Cryptococcus neoformans*, and *Aspergillus* sp. are responsible for high mortality especially in immunocompromised individuals and pwCF (87, 88). However, the impact of bioenergetic pathways during fungal infection is still an emerging field, and how it associates with clinical outcomes remains unclear.

**Figure 4:**
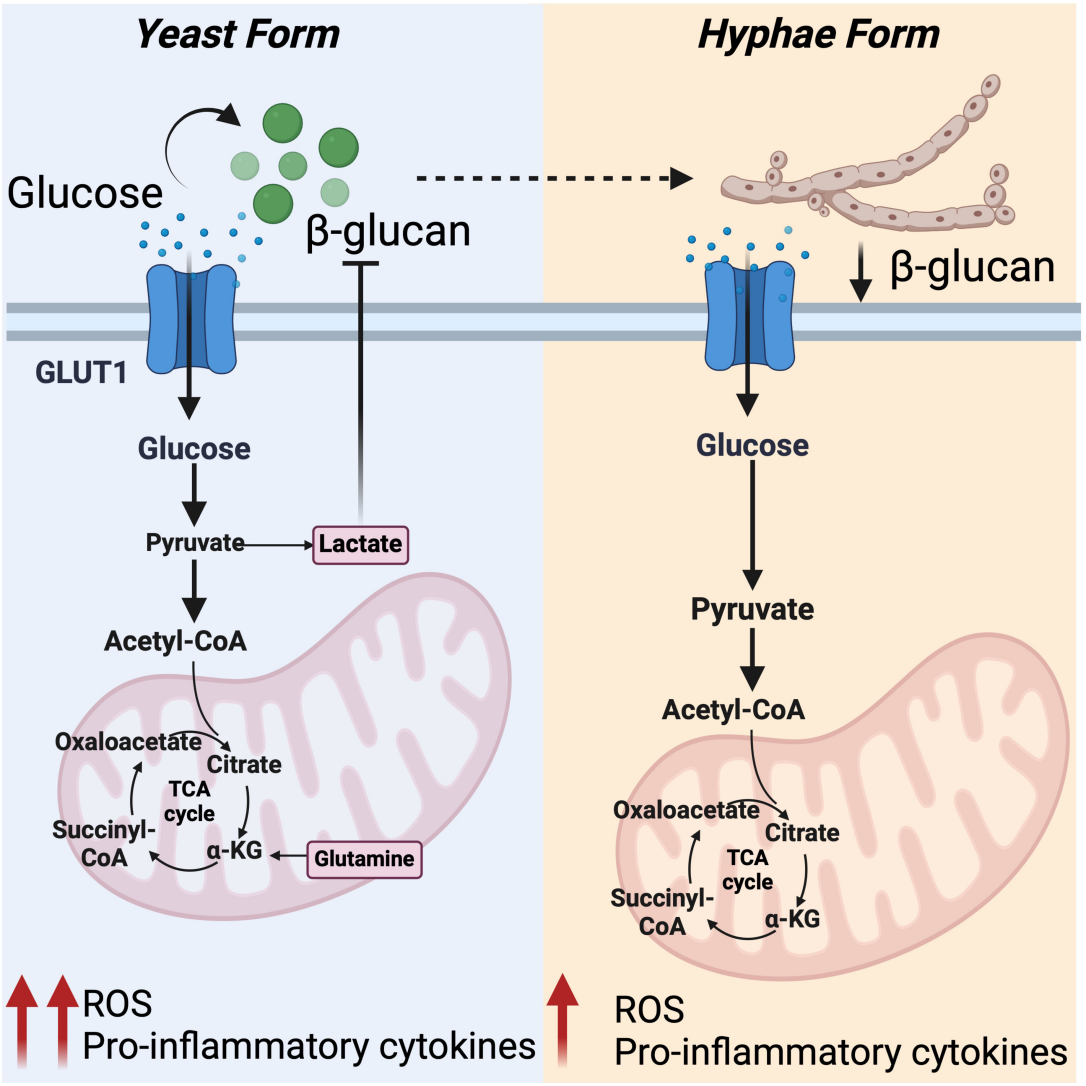
Differential regulation of host metabolic pathway by *C. albicans*: Left panel: In response to *C. albicans* β-glucans, primarily exposed on yeast form, host cell responds by enhancing glycolysis, OXPHOS, and glutamine metabolism. This metabolic shift triggers anti-fungal responses by supporting ROS production and cytokine response. Enhanced glycolysis results in elevated lactate levels, which limits β -glucans expression and drives glycolysis in *C. albicans*, depleting glucose from extracellular milieu. Right panel: Reduced exposure of β -glucans on hyphae form of *C. albicans* fails to activate glycolysis and the pentose phosphate pathway, leading to reduced mitochondrial ROS production and cytokine response. This setting favors *C. albicans* survival.

Recent findings indicate that glycolysis is central to the immune response against fungal infection (89, 90). Anti-fungal neutrophils rely heavily on glycolysis, particularly on expression of key glucose transporters, like GLUT1 (90). GLUT1 perturbation in these cells blunt phagocytosis, ROS, and formation of extracellular traps, facilitating fungi survival (89, 90). During *C. albicans* infection, in the yeast phase, host cells respond by promoting glycolysis, OXPHOS, and glutamine metabolism. This supports higher energy demands required for a robust oxidative burst and cytokine response. However, at later stages, as *C. albicans* transitions from yeast to hyphal forms, the induction of glycolysis and OXPHOS is reduced leading to an attenuated oxidative burst and a diminished cytokine response (91). These distinctive programs are attributed to the difference in the cell wall composition, such as the presence of β-glucans, that acts as a major PAMP for the PRR Dectin-1 (92). To curb host immunity, *C. albicans* reduces the surface expression of β-glucans, particularly when exposed to the glycolysis byproduct lactate (93) (**Figure 4**). This adjustment also promotes active glycolysis in *C. albicans*, depleting glucose form the milieu and starving immune cells to death (94). Inhibition of host glycolysis by 2-DG or by dichloroacetate, which is an enhancer of pyruvate dehydrogenase, perturbs the host response against *C. albicans*. This phenotype is associated with reduced expression of IL-1β, TNFα, and IL-6 in human monocytes (91, 95). Additionally, these treatments impair the production of IL-17, IL-22, IFN-γ and IL-10, cytokines regulated by Th1/Th17 T cells and crucial for host protection during *C. albicans* infection (91). In line with this, activation of host glycolysis by nutritional supplementation of glucose facilitates health and *C. albicans* eradication (94). Thus, during *C. albicans* infection, there is a dynamic host-pathogen glycolytic competition that determines the outcome of the disease.

In contrast to *C. albicans*, melanin is a major component of the cell wall in *A. fumigatus (* 96 *)*. This melanin serves as a key driver of metabolic reprogramming in immune cells (97). Mechanistically, within the phagosome, melanin released from the spores promotes the sequestering of calcium, which triggers the recruitment of mTOR on the phagosome (97). mTOR is a major regulator of glycolysis. It regulates the expression level of HIF1α and other genes involved in glycolysis (98). By promoting mTOR and HIF1α activity, *A. fumigatus* induces glycolysis in the infected macrophage, leading to the release of cytokines involved in tissue oxidation (97). Additionally, melanin also aids in the consolidation of the pathology by modulating calcium signaling. Melanin effectively blocks secretion of chemokines such as CXCL1 and CXCL8, reducing the infiltration of anti-fungal immune cells (99). These studies show how fungal pathogens use specific PAMPS to modulate glycolysis, cytokine release, and myeloid cell recruitment. By stimulating glycolysis, fungal organisms trigger inadequate immune responses, leading to tissue disruption and organ disease.

## Conclusion

In this review, we highlight the impact of metabolism on host-pathogen interactions in the lung, particularly through modulation of cellular bioenergetics. We examine recent evidence suggesting how pulmonary pathogens exploit host bioenergetic platforms to persist, and how this environment influences inflammatory responses and disease progression. Bacterial pathogens like *P. aeruginosa*, Mtb, and *S. aureus* alter lung cell bioenergetics - inducing shifts like increased glycolysis, impaired OXPHOS, and the release of metabolites like succinate, proline, and itaconate - to evade immune responses and promote survival. This metabolic disruption weakens antimicrobial defenses, exacerbates tissue damage, and drives chronic inflammation. Although similar findings have been reported during fungal diseases, the influence of bioenergetics and metabolism on mycoses remains underexplored. Metabolism plays a critical role in many physiological processes, which can be co-opted by opportunists to drive tissue disruption via a range of mechanisms, including autophagy, cell death pathways – i.e., apoptosis and ferroptosis - and epigenetic modifications, shaping immune responses to fuel pathology evolution instead of organ repair. Given the impact of bioenergetic platforms on infection outcomes, these metabolic pathways emerge as potential therapeutic targets for drug design. Key players such as GLUT1/3, HK, and SDH have been considered to prevent exacerbated inflammatory response and promote anti-inflammatory routes, especially during sepsis and other immunopathologies (17). Similarly, inhibition of PKM2 (Pyruvate Kinase M2) during sepsis reduces inflammation via NLRP3 (100). This also provides implication of nutrition in bioenergetics dynamics. However, further investigation is needed to uncover how dietary interventions, or metabolic modulators could influence infectious disease progression. Interfering pathogens from utilizing immunometabolites such as succinate, itaconate, and lactate could also aid in clearing the infection and resolution of inflammation. On the host side, major challenges associated with immunometabolism-based therapy are the so-called off-target effects, impacting homeostasis of “healthy” cells. In such scenarios, delivering the drugs to the specific cells by utilizing nanoparticle formulation would improve treatment outcome (17, 101). These processes may open new avenues for metabolo-therapies aimed at restoring immune balance, mitigating mucosal damage, and limiting pathogen persistence in diseases such as pneumonia.
